# A Novel Lightweight Dairy Cattle Body Condition Scoring Model for Edge Devices Based on Tail Features and Attention Mechanisms

**DOI:** 10.3390/vetsci12090906

**Published:** 2025-09-18

**Authors:** Fan Liu, Yongan Zhang, Yanqiu Liu, Jia Li, Meian Li, Jianping Yao

**Affiliations:** College of Computer and Information Engineering, Inner Mongolia Agricultural University, Hohhot 010018, China; lfmw@emails.imau.edu.cn (F.L.); liuyq@imau.edu.cn (Y.L.); lijia@imau.edu.cn (J.L.); 20071036@imau.edu.cn (M.L.); yaojianping@emails.imau.edu.cn (J.Y.)

**Keywords:** tail features, computer vision, lightweight model, model distillation, edge computing

## Abstract

**Simple Summary:**

This study introduces a new method for assessing the physical condition of dairy cows using a lightweight deep learning model, which achieves this by analyzing tail. The traditional methods are time-consuming and subjective, and are not suitable for large-scale farming. The proposed model utilizes computer vision and machine learning technologies to automatically assess the physical condition of dairy cows by focusing on tail features. It performs well on resource-constrained edge devices and is highly suitable for real-time farm monitoring. The model achieves high accuracy in assessing the health status of dairy cows, significantly improving processing speed and storage efficiency. This research provides a practical solution for improving livestock health management and promotes the development of intelligent livestock farming technology.

**Abstract:**

The Body Condition Score (BCS) is a key indicator of dairy cattle’s health, production efficiency, and environmental impact. Manual BCS assessment is subjective and time-consuming, limiting its scalability in precision agriculture. This study utilizes computer vision to automatically assess cattle body condition by analyzing tail features, categorizing BCS into five levels (3.25, 3.50, 3.75, 4.0, 4.25). SE attention improves feature selection by adjusting channel importance, while spatial attention enhances spatial information processing by focusing on key image regions. EfficientNet-B0, enhanced by SE and spatial attention mechanisms, improves feature extraction and localization. To facilitate edge device deployment, model distillation reduces the size from 23.8 MB to 8.7 MB, improving inference speed and storage efficiency. After distillation, the model achieved 91.10% accuracy, 91.14% precision, 91.10% recall, and 91.10% F1 score. The accuracy increased to 97.57% for ±0.25 BCS error and 99.72% for ±0.5 error. This model saves space and meets real-time monitoring requirements, making it suitable for edge devices with limited resources. This research provides an efficient, scalable method for automated livestock health monitoring, supporting intelligent animal husbandry development.

## 1. Introduction

Body Condition Scoring (BCS) is one of the important indicators for measuring the health status of cows or other livestock. It is commonly used to evaluate their fat reserves and energy metabolism levels. Moreover, BCS is of great significance for health management, reproductive efficiency, and production performance in cows [1]. Research has shown that BCS is valuable in the prevention of mastitis, reproductive health monitoring, control of metabolic diseases, and enhancement of immunity and disease resistance [2,3,4]. Abnormal BCS can significantly affect postpartum recovery and the health of cows. Low body condition may lead to postpartum diseases such as endometritis and mastitis, while high body condition can lead to ketosis or fatty liver [5]. Therefore, scientific and reasonable BCS management plays a crucial role in improving the health and breeding efficiency of dairy cows.

The traditional BCS assessment mainly relies on manual visual scoring [5,6]. Usually, professional assessors make judgments by observing the fat distribution in areas such as the cow’s dorsal line, ischial tuberosity, and tail root. This method is subjective and has different evaluation criteria, making it difficult to meet the needs of modern farms for efficient, standardized, and automated management [7,8,9,10].

With the development of computer vision and deep learning technologies, automated evaluation methods for BCS have received widespread attention. Bewley and Schutz reviewed the multidisciplinary applications of BCS in dairy cow management, emphasizing the important role of image analysis in improving BCS accuracy [2]. Goselink et al. [11] indirectly demonstrated the potential of automated monitoring methods in exploring the metabolic adaptability of dairy cows. Dandıl et al. [12] designed an automated classification system based on the YOLO architecture, achieving an 81% classification accuracy on multiple cow breed images, demonstrating the practical potential of BCS automatic evaluation.

In recent years, deep learning has made significant progress in the field of dairy cow BCS. However, the existing BCS methods generally face problems of high computational complexity and low real-time performance, which limits their application on edge devices. There are always imbalances in the three aspects of classification accuracy, model size and operational efficiency. This paper aims to address the issues currently faced in specific agricultural applications through a lightweight model, and the proposed model is precisely designed to fill this gap.

TinyML (Machine Learning based on Ultra-Low Power Microcontrollers) has become an important research direction for achieving intelligence on edge devices. Relevant studies have explored how to combine TensorFlow Lite with ultra-low power microcontrollers such as Arduino to implement efficient machine learning applications, especially the advantages in handling low-power devices [13,14]. Moreover, in recent years, research on the combination of edge computing and artificial intelligence has also made significant progress, particularly in the implementation of device-level intelligence. Researchers have explored the integration of edge computing and artificial intelligence to provide support for low latency and high performance [15].

In the fields of agriculture and veterinary medicine, the combination of computer vision and deep learning has significantly promoted the development of precision agriculture. Relevant studies have deeply explored the application of computer vision in tasks such as crop disease detection, weed identification, and crop classification, demonstrating the significant role of this technology in improving agricultural efficiency and accuracy [16,17]. Particularly in the areas of plant disease detection and weed identification, researchers have continuously improved deep learning models, enhancing the accuracy of the algorithms and their ability to identify different crop diseases [18,19].

In the field of animal science, computer vision technology is gradually being applied to aspects such as animal health monitoring and behavior analysis. Convolutional Neural Networks (CNN) are widely used in animal agriculture, especially in the automatic identification of animal behaviors and health monitoring, helping to enhance the automation and accuracy of agricultural production [20,21]. Moreover, the combination of computer vision and generative models in agriculture has also promoted further technological innovation, especially in the intelligent application of livestock management and agricultural production systems [22,23]. All of these provide us with a feasible solution.

For instance, the method proposed by Shao et al. for detecting key parts of dairy cows based on the improved You Only Look Once version 5 (YOLOv5) algorithm effectively improved the scoring efficiency, but it still needs further optimization to meet the requirements of real-time processing in more complex environments [24]. The scoring system proposed by Lewis et al. [25], based on 2D images and deep learning, although it improved the accuracy, had a large computational cost and was difficult to run on resource-constrained devices. The lightweight convolutional neural network proposed by Feng et al. [26] improved computational efficiency and reduced model complexity; however, it still failed to meet the requirements of large-scale data processing and high-resolution images, particularly in edge computing environments.

To address this issue, Li et al. [27] proposed an improved YOLOv5 model that combines video analysis and is adapted to score the body condition of cows in a group setting. However, it still faces challenges in real-time processing on edge devices. Additionally, scoring methods based on 3D images and depth images, such as the multi-camera scheme proposed by Li et al. [28] and Summerfield et al. [29], although they improve the scoring accuracy, are unable to be widely applied in resource-constrained environments due to the large amount of data processing and the reliance on high-performance hardware.

The scoring method proposed by Lee et al. [30], which combines deep neural networks and 3D imaging, effectively improved the scoring accuracy, but it required high computing resources and still faced challenges in real-time processing. Luo et al. [31] proposed a lightweight convolutional neural network and channel attention mechanism that performed well in pig posture detection, but still encountered limitations due to hardware and computing capabilities when dealing with complex scenarios. Meanwhile, Yukun et al. [32] proposed an automatic monitoring system based on a deep learning framework, which achieved BCS through identification of dairy cows’ body parts, although it had great potential, it still faced challenges in large-scale and real-time monitoring.

Furthermore, the deep image analysis and annotation optimization methods proposed by Li et al. [28] and Nagy et al. [33], although they can improve the accuracy of BCS, still have high requirements for hardware performance when dealing with large-scale data, which limits their application in real-time edge computing.

Despite significant progress in existing research, the robustness and adaptability of current methods still need to be improved, especially in complex environments. Furthermore, some deep learning models come with high computational and storage costs, which makes them difficult to deploy in resource-constrained on-site settings. For instance, YOLOv8n has a relatively heavy computational load in complex tasks, which may affect the efficiency of its operation [34]. Additionally, their real-time performance remains limited, restricting their practical application in large-scale aquaculture farms.

In studies on other animal BCS and posture detection, many works have adopted similar tail features as important visual input features. For instance, Huang et al. [35] used the Faster R-CNN (Region-based Convolutional Neural Network) method to detect the tails of dairy cows in their research. It combines the Region Proposal Network (RPN) and the Convolutional Neural Network (CNN) to enhance the efficiency and accuracy of object detection, aiming to provide a basis for BCS. Nagy et al. [33]’s study also demonstrated the application of deep learning in dairy cow BCS, particularly through the analysis of images in the hip area, exploring the influence of classification categories and annotation areas on the prediction of BCS. Based on existing experience and research observations, it has been found that the BCS of dairy cows changes. In the BCS, tail characteristics refer to the morphological changes in the cow’s tail obtained through image analysis, which specifically include the shape of the tail, the degree of depression on both sides of the tail root, and the degree of expansion and flatness of the tail. With the change in BCS, these tail characteristics will undergo significant changes. For example, the tails of cows with poor body condition may show larger depressions, while the tails of cows with better body condition are flatter or slightly expanded. Different from the traditional method of scoring using the entire cow’s image, tail characteristics can more accurately reflect the fat distribution and health status of the cow by focusing on the local details of the cow’s tail. This makes tail characteristics an efficient and practical assessment standard, which, especially when visual information is relatively clear, can reduce errors and improve the accuracy of the score. Therefore, the tails of dairy cows will be used as the standard for BCS in this paper.

To address these challenges, this paper proposes a lightweight cattle condition scoring model based on the EfficientNet [36,37,38] architecture. The model integrates the SE channel attention module (Squeeze and Excitation) [39], spatial attention module [40], label smoothing [41], and a YOLOv5 classification head [42,43,44] to provide real-time and accurate cattle condition scoring. The design aims to balance classification accuracy, model size, and operational efficiency. The inclusion of both channel and spatial attention mechanisms enhances the model’s ability to focus on key feature areas and improves its robustness in complex environments. Label smoothing strengthens classification stability and helps alleviate overfitting. The YOLOv5 classification head simplifies the multi-class classification structure, thereby boosting the model’s generalization ability in multi-class recognition tasks. This solution significantly reduces the model’s computational complexity and memory usage, while maintaining high accuracy, making it suitable for application in grazing systems.

## 2. Materials and Methods

### 2.1. Selection of Dataset

**Figure 1:**
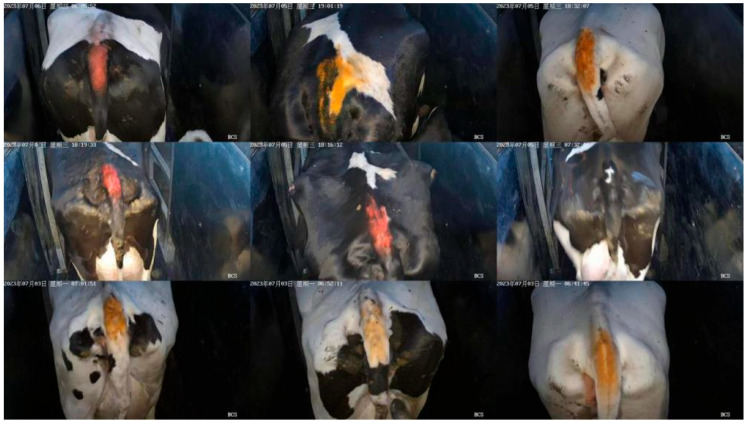
Example of Cowtail Image Dataset.

The dataset used in this experiment is a publicly available dataset from ScienceDB [45], mainly used to evaluate the production performance and physical health status of cows. This dataset contains 53,566 images, and some examples of the dataset are shown in Figure 1. Collected from Lu’an City in Anhui Province, Huai’an City in Jiangsu Province, and Wuwei City in Gansu Province. All images are from large-scale breeding farms, and each image is rated by a dedicated team to ensure consistency and accuracy of labeling. This study carried out basic preprocessing: the images were cropped based on the annotated tail regions, and uniform adjustments were made to ensure consistent input sizes. Due to the large size of the dataset, and the fact that it already contains rich diversity, including different lighting conditions, backgrounds, and the postures of the cattle. This inherent diversity enables the model to directly learn robust features from the original data distribution. Scored using the standard BCS scoring method [1].

A detailed preprocessing of the data was first conducted, including filling in missing values, handling outliers, and normalizing features. The dataset was divided into training, validation, and test sets to ensure the generalization ability of the model. For model training, the EfficientNet deep learning model was selected, and the hyperparameters (such as learning rate, batch size, etc.) were optimized through grid search. During the training process, the Adam optimizer was used, and appropriate training epochs and early stopping strategies were set to avoid overfitting. The model performance was evaluated using metrics such as accuracy, precision, recall, and F1 score, and compared with baseline models (such as logistic regression or decision tree), demonstrating a significant improvement in performance of the proposed model.

In this study, “tail characteristics” refer to the features of the tail area of dairy cows. These characteristics are closely related to the fat distribution, health status, and BCS of the cows. The tail morphology features include the overall outline of the tail, the degree of depression at the base of the tail, and the flatness or expansion state of the tail. Cows with poor body condition may have a significantly depressed base of the tail, while cows with better body condition show a more flat or slightly expanded tail shape. These morphological changes reflect the fat distribution of the cows, and thus can be used to infer the BCS of the cows.

Each image in the dataset is labeled with a cattle BCS area, which is divided into 5 rating categories based on BCS from lean to fat: 3.25, 3.5, 3.75, 4.0, and 4.25. Different ratings reflect the different health conditions and production performance of cows.

**Figure 2:**
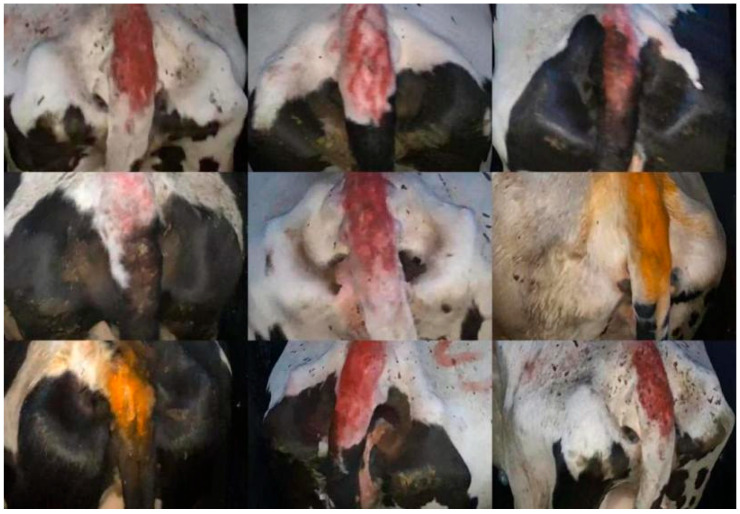
Example of Cowtail Image Segmentation Dataset.

In this experiment, the dataset was divided into a training set, validation set, and testing set in a ratio of 7:2:1. The images were batch-cropped based on their corresponding annotations. The initial image has been labeled. The area of the cow’s tail was cropped and extracted using a custom script for labeling. The processed effect is shown in Figure 2. The training set consists of 37,496 images (70% of the total dataset), the validation set consists of 10,713 images (20% of the total dataset), and the test set consists of 5357 images (10% of the total dataset).

### 2.2. Test Environment

The experiment was conducted on a computer equipped with an RTX 4090 (24 GB) graphics card, which provides powerful computing power for deep learning tasks. In addition, the system is equipped with a 16 core Intel Xeon Gold 6430 processor and 120 GB of memory. The system disk is 30 GB, and the data disk provides 50 GB of storage space for storing training data and intermediate results. The operating system is Ubuntu 22.04, developed using Python 3.12, and the deep learning framework is PyTorch 2.5.1, combined with CUDA12.4 to fully utilize GPU accelerated training.

## 3. Results

### 3.1. Selection of Benchmark Network Model

This article takes lightweight models as the research object. Firstly, six classic light models were experimentally compared and evaluated using a public cow dataset. These models include: EfficientNet-B0, MobileNetV3-Large [46], MobileNetV2 [47], MobileNetV3-Small, SqueezeNet-1 [48], and ShuffleNet [49]. The above model is widely used in mobile visual recognition tasks due to its compact structure, low parameter count, and suitability for edge deployment. In this study, we used it as a candidate baseline model to comprehensively evaluate its performance under multidimensional performance indicators.

#### 3.1.1. Confusion Matrix of Benchmark Network Model

**Figure 3:**
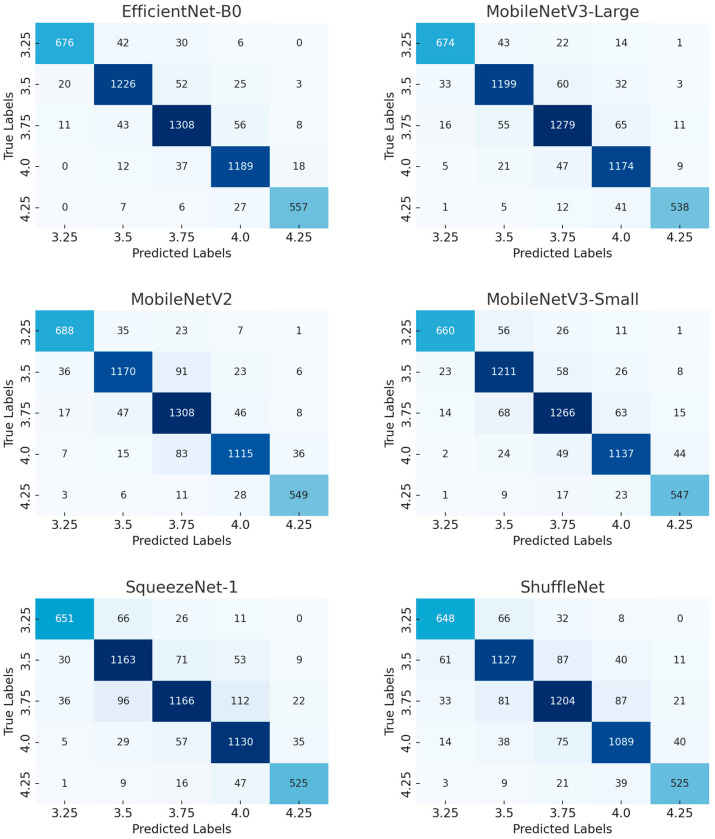
Comparison of confusion matrices for basic models. This figure presents the confusion matrices of different models, with each small graph representing the classification result of a specific model. The shade of color in the figure reflects the prediction accuracy: darker colors (dark blue) indicate that the model is more accurate in that category, with a higher number of correctly classified samples; while lighter colors (light blue) indicate that there are more misclassified samples, and the model performs poorly in these categories. The values in each matrix represent the number of samples predicted to belong to a certain category. The x-axis represents the predicted label, and the y-axis represents the actual label.

Figure 3 presents the confusion matrices of the selected six base models. Each model’s confusion matrix shows its performance in different BCS classifications. Through these confusion matrices, the performance differences in each model across different score ranges can be visually observed.

From these charts, it can be seen that EfficientNet-B0 performs the best in terms of accuracy, with relatively precise predictions for almost all categories. Especially in the lower BCS (such as 3.25 and 3.5) categories, there are fewer incorrect classifications. In contrast, ShuffleNet and SqueezeNet-1 perform poorly in most categories, especially in the cattle body type classified as 4.25 (a higher BCS), where incorrect classifications are more severe. Incorrect classifications usually occur in adjacent BCS categories (such as between 3.75 and 4.0), which may indicate the limitations of these models in handling subtle differences.

Furthermore, from the data in the figure, it can be seen that MobileNetV3-Large and MobileNetV3-Small have experienced more confusion when processing some high-score (such as 4.25) and low-score (such as 3.25) cattle bodies. This indicates that although these models are relatively light in terms of computation, their performance may be limited by the simplicity of their model structure in certain complex scenarios.

Overall, EfficientNet-B0 has an advantage in balancing accuracy and recall, and has fewer misclassifications, making it suitable for tasks with high accuracy requirements. Other models, such as SqueezeNet-1 and ShuffleNet, may require further optimization or integration of more powerful feature extraction modules to improve their performance.

**Figure 4:**
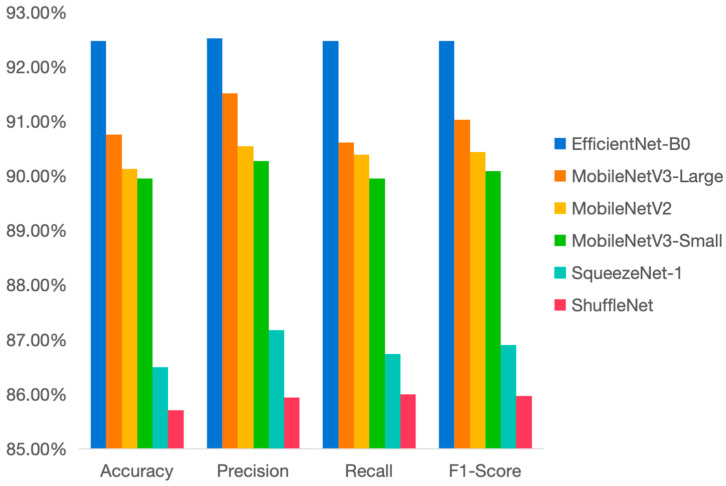
Comparison of Basic Model Charts.

Figure 4 shows the performance of different models in terms of accuracy, precision, recall rate, and F1 score. According to the chart, EfficientNet-B0 demonstrates the best overall performance, particularly in terms of accuracy (about 92.48%), precision (92.53%), and F1 score (92.48%), and becomes the selected benchmark model. In contrast, ShuffleNet shows the worst performance, with all indicators significantly lower than those of other models. Among them, the accuracy rate is 85.71%, which is much lower than that of other models.

#### 3.1.2. Comparative Experiment of Basic Model

This experiment introduced multi-dimensional indicators to systematically evaluate the performance of the model, including conventional classification performance indicators (accuracy, precision, recall, F1 score) expressed as percentages, model structure indicators (model size in megabytes, million floating-point operations, million parameters), inference efficiency indicators (delay in milliseconds), and fault tolerance evaluation indicators set for BCS-level classification tasks (accuracy ± 0.25 and ±0.5), to comprehensively test the accuracy, efficiency and fault tolerance capabilities of the model in practical applications. As shown in Table 1.

From the overall experimental results, EfficientNet-B0 leads the six lightweight models with an accuracy of 92.48%, an accuracy of 92.53%, a recall rate of 92.48%, and an F1 score, making it the optimal candidate for the backbone network in this study. The accuracy of the BCS under error tolerances of ±0.25 and ±0.5 reached 97.98% and 99.70%, respectively, demonstrating high-level differentiation ability and fault tolerance performance.

Although the model volume of EfficientNet-B0 is 15.31 MB and the FLOPs reach 400.39 M, its computational cost is slightly higher than models such as MobileNetV2 (8.7 MB, 312.92 M FLOPs) and SqueezeNet (2.8 MB, 263.06 M FLOPs). However, it still maintains a good level of inference delay (0.6829 ms), which is better than MobileNetV3 Large (0.9772 ms) and V2 (0.9386 ms). In contrast, although ShuffleNet has the smallest model size (5 MB) and lower computational overhead (147.79 M FLOPs), its accuracy is only 85.71% and F1 score is 85.97%, indicating lower overall performance.

### 3.2. Improvement of Benchmark Network Model

In this study, a series of optimizations were carried out on the EfficientNet-B0 model to improve its small object detection accuracy and robustness in cattle tail images. Small targets in cow tail images usually have low resolution and low contrast with the background, so traditional object detection models perform poorly in such images. In order to improve the accuracy of model recognition, this study proposes the following improvement measures for the basic EfficientNet-B0 model.

There are four sets of comparative experiments in this section, using the basic EfficientNet-B0 model combined with the SE Attention channel attention module, Spatial Attention module, Label Smoothing Loss, and YOLO classification head to detect performance improvement in the newly added model. In the evaluation process, the classification performance, structural complexity, and inference efficiency of the model were comprehensively considered. Specifically, it includes indicators such as accuracy, precision, recall, and F1 score to measure classification ability; complexity metrics such as model size, floating point operations (FLOPs), and number of parameters; and the average inference latency of a single image is used as an efficiency evaluation. At the same time, to verify the discriminative ability of the model in handling boundary-level samples, additional BCS tolerance accuracies of ±0.25 and ±0.5 are introduced as fault tolerance evaluation indicators.

#### 3.2.1. Introducing SE Attention Channel Attention Module

SE attention is a channel attention mechanism that extracts global features of each channel through global average pooling, generates importance weights for each channel through two fully connected layers, and finally performs weighted recalibration between channels on the original feature map to enhance the model’s ability to focus on key features

**Figure 5:**
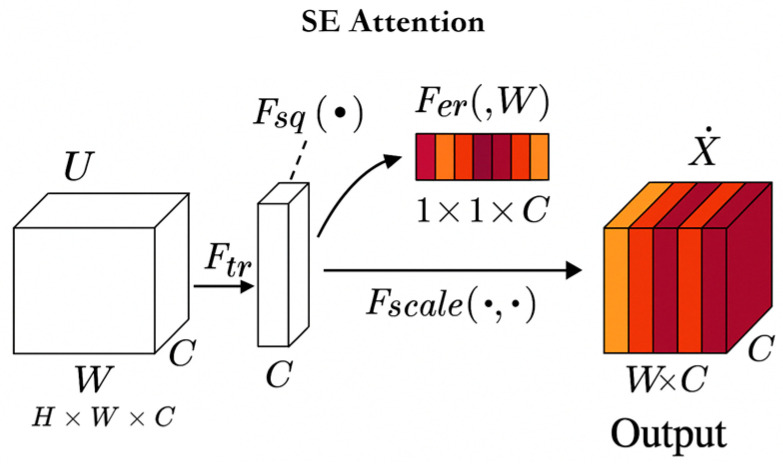
SE attention mechanism. The SE Attention module in the figure adjusts the influence of each feature by weighting the input features. The shade of color indicates the weight of the feature. Darker colors represent that the feature is given a higher weight in the attention mechanism and has a stronger influence, while lighter colors indicate that the feature has a lower weight and contributes less to the final result. This module enables the model to focus more on important features by adaptively adjusting the weights of feature channels, thereby improving the model’s performance.

In order to enhance the model’s perception ability of key features, the SE Attention module was introduced in the EfficientNet-B0 model, as shown in Figure 5. SE Attention can enhance the perception ability of the base model on the fat layer features of the cow tail root by adaptively adjusting the weights of each channel, while also improving the ability to suppress environmental noise (such as changes in lighting).

Equation (1) represents that in the SE module, the input feature map F_input is first subjected to a global average pooling operation in the channel dimension to obtain the feature description vector F_sq. This vector sequentially passes through two fully connected layers (corresponding to weights W_1_ and W_2_), and applies ReLU activation function δ and Sigmoid activation function σ, respectively, to generate channel-level weight coefficients. Finally, by multiplying with the original input feature map F_input, a weighted output feature map F_scale is obtained, thereby achieving explicit modeling and enhancement of key channel information.

(1) $$F\_scale=F\_input\times \sigma (W_{2}\;\cdot \;\delta (W_{1}\;\cdot \;F\_sq))$$

The classification performance improvement effect of the introduced model is shown in Table 2. The increase in amplitude is given in percentage terms.

After introducing the SE attention mechanism, the model achieved slight improvements in multiple key indicators. Among them, the accuracy increased from 92.48% to 92.55%, and the F1 score increased from 92.48% to 92.56%. Under the BCS ± 0.25 tolerance evaluation, the accuracy increased by 0.04 percentage points, indicating an enhanced ability to distinguish between adjacent levels (such as BCS3 and BCS4). Although the number of parameters slightly increased (+0.21 M) and the model volume increased to 16.38 MB, the inference delay was actually shortened by 0.0406 ms, indicating that the SE module did not significantly increase the inference burden while improving the representation ability, but instead optimized the computational path efficiency. The SE module enhances the feature modeling ability between channels while maintaining the overall slim structure of the model, which is an effective structural improvement worth adopting in practical deployment.

#### 3.2.2. Introducing the Spatial Attention Module

The Spatial Attention module compresses the channel feature map (usually using average pooling and max pooling), and then convolves to extract spatial weight maps, thereby weighting each position of the input feature map and enhancing the model’s attention to key spatial regions

**Figure 6:**
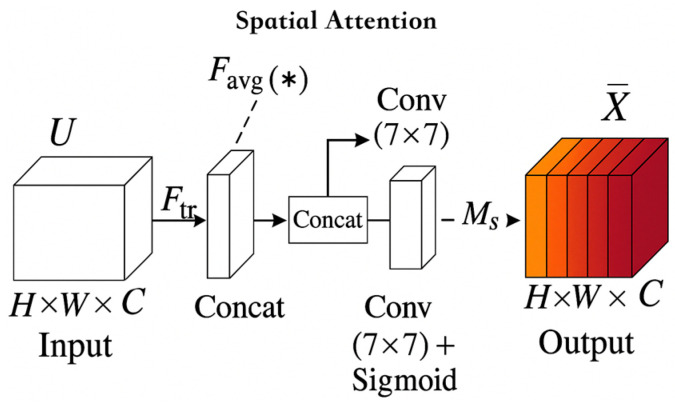
Spatial Attention Module. The shades of color in the figure represent the attention weights of different spatial regions in the spatial attention mechanism. Darker colors indicate that the model pays more attention to that region, while lighter colors indicate lower attention. F_avg_(*) represents the average pooling operation on the input features. By aggregating information in the spatial dimension, it helps the model capture more global features. This mechanism enhances the model’s attention to important regions by weighting the features of different regions, thereby improving the model’s performance.

The Spatial Attention module generates spatial-level attention maps and weights different regions of the image, enabling the base model to enhance the weight of the tail root region in cattle condition scoring tasks, ignoring irrelevant information such as hair texture and background noise, and enhancing the model’s spatial information processing capability. As shown in Figure 6.

Equations (2) and (3) represent that the Spatial Attention module first performs channel wise average pooling and max pooling operations on the input feature map F, obtaining two types of spatial attention cues. Subsequently, these two feature maps are concatenated in the channel dimension, and fused features are extracted through a 7 × 7 convolutional layer. The spatial attention map M_s_ (F) is then normalized using the Sigmoid function. Finally, the attention map is multiplied element by element with the original feature map F to obtain a weighted output F_scale, thereby enhancing the model’s responsiveness to key spatial regions and suppressing irrelevant information.

(2) $$M_{s}(F)=\sigma (Conv(7\times 7)(Concat[AvgPool(F);\;MaxPool(F)]))$$

(3) $$F\_scale=M_{s}(F)\times F$$

Table 3 is a comparison table between the basic model and the separately introduced Spatial Attention module

After introducing Spatial Attention, the overall performance of the model was further improved, with accuracy increasing from 92.48% to 92.82% and F1 score increasing from 92.48% to 92.82%. All indicators achieved an improvement of approximately 0.34 percentage points. Especially within the BCS ± 0.25 tolerance range, the accuracy increased from 97.98% to 98.04%, indicating that the spatial attention mechanism effectively enhances the model’s perception ability of local key areas and helps identify subtle level differences. In terms of computational cost, although the number of parameters slightly increased (+0.07 M), FLOPs increased by about 3 M, and the model volume increased to 15.84 MB, the overall change was small, and the inference delay only slightly increased by 0.0234 ms, which is still acceptable. Overall, Spatial Attention significantly improves the model’s discriminative and fine-grained perception capabilities while maintaining its slim characteristics, making it suitable for high-precision recognition tasks such as BCS in animal husbandry vision.

#### 3.2.3. Introducing Label Smoothing Loss

Label smoothing loss improves generalization ability by smoothing the original “hard labels” (such as one hot encoding) into “soft labels”, introducing a small amount of uncertainty to prevent overfitting of the model to the training data.

**Figure 7:**
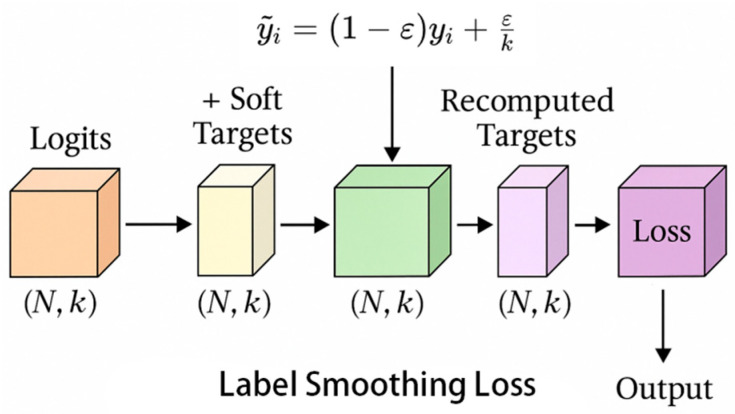
Label smoothing loss module.

In order to further enhance the recognition ability of the model for key regions in images, the Spatial Attention module was introduced into the model, as shown in Figure 7. This module generates spatial-level attention maps and weights different regions of the image, allowing the model to focus more on the target area, especially for capturing small targets, enhancing the model’s spatial information processing capabilities.

The Label Smoothing Loss function, as shown in Equation (1), was used to prevent overfitting by reducing the model’s overconfidence in class labels, particularly in imbalanced or noisy datasets, thereby improving generalization.

In Label Smoothing, *y*_i_ represents the one hot encoding of the real label, *p*_i_ represents the probability of the model predicting it as class i, *ε* is the smoothing coefficient that controls the degree of softening of the label distribution, usually taken as 0.1, and *K* is the total number of classifications. The smoothed label distribution is shown in Equation (4), where *ỹ*_i_ represents the target distribution obtained through the smoothing operation. The final loss function is Equation (5), which is used to replace the cross-entropy loss function of the original one hot encoding to alleviate overfitting problems and improve the model’s generalization ability.

(4) $$\tilde{y_{i}}=\left(1-\epsilon \right)\cdot y_{i}+\frac{\epsilon }{K}$$

(5) $$L_{L}=-\sum_{i=1}^{K}\tilde{y_{i}}\cdot \log\left(p_{i}\right)$$

The classification performance improvement effect of the introduced model is shown in Table 4.

After introducing Label Smoothing, the classification performance of the model has achieved stable improvement in multiple key indicators. The accuracy has increased from 92.48% to 92.70%, and the F1 score has also increased from 92.48% to 92.71%, with an overall improvement of approximately 0.22–0.23 percentage points. Under the BCS ± 0.25 tolerance evaluation, the accuracy improvement was more significant, increasing from 97.98% to 98.28%, indicating that label smoothing effectively alleviated the overfitting of the model to a single label and enhanced the recognition ability of fuzzy samples between adjacent levels (such as BCS3 and BCS4). From the perspective of computing resources, although the introduction of this mechanism resulted in an increase of 13.48 M in FLOPs, a growth of approximately 1.09 MB in model volume, and a slight increase in inference delay to 0.7124 ms, the number of parameters remained unchanged, and the overall resource cost remained within an acceptable range. In summary, Label Smoothing can effectively improve the generalization ability and classification robustness of the model without increasing its complexity, making it a practical and deployment friendly optimization strategy.

#### 3.2.4. Introducing YOLOv5 Classification Header

The classification head of YOLOv5 is a lightweight convolutional structure that receives the output from the feature pyramid and outputs the probability distribution of the category for each predicted box position (usually achieved through a sigmoid or softmax activation function).

**Figure 8:**
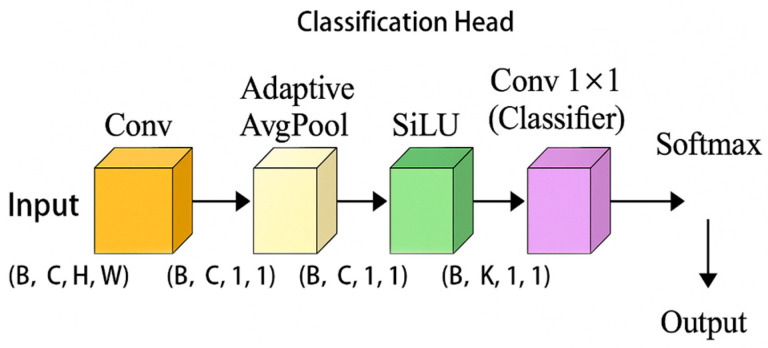
YOLO classification head.

In the model, YOLO classification header is used, as shown in Figure 8, to improve the performance of classification tasks. The YOLO classification head has been optimized and designed to perform target classification more efficiently, especially when dealing with small targets and complex backgrounds. The performance of the classification head can effectively improve the overall detection accuracy. Through this classification header, the model can quickly and accurately classify the detected recognition targets.

The introduction of the YOLO classification head in this study aims to replace the traditional fully connected classification layer. The fully connected layer is commonly used for multi-class classification from feature maps, but this method often increases the computational load, especially when performing inference on resource-constrained devices, which may lead to performance bottlenecks. Through multiple strategies, the efficiency and classification accuracy of the minimal architecture have been significantly improved. Firstly, depthwise separable convolution was adopted to replace the traditional convolution operation. Depthwise separable convolution splits the convolution operation into channel-wise convolution and point-wise convolution, significantly reducing the number of parameters and computational load, thereby reducing memory consumption and inference latency. Additionally, to further alleviate the computational burden, the YOLO classification head adopted a light convolution structure, simplifying the design of the fully connected layer and avoiding the problems of a large amount of computation and storage overhead in traditional fully connected layers.

The model can focus on the key areas of the image when dealing with complex scenes, especially in the detection of small targets, such as tail areas. Traditional target detection models often perform poorly in the recognition of small targets, while the YOLO classification head significantly improves the classification accuracy of small targets by enhancing local perception and feature reuse capabilities. These optimizations not only improve the model’s performance in different scales and complex backgrounds, but also ensure its adaptability and efficiency, especially in real-time inference applications on edge devices. Ultimately, these optimization measures effectively balance classification accuracy, model size, and inference speed, ensuring feasibility and efficient performance in resource-constrained environments.

In the cross entropy loss function Equation (6), yᵢ represents the one hot encoding of the true label, pᵢ represents the prediction probability of the model for the i-th class, and K is the total number of classes. This formula measures the closeness between the model output and the true label by calculating the distance between the predicted distribution and the true distribution. The closer the predicted result is to the true label, the smaller the loss value.

(6) $$L=-\sum_{i}\;y_{i}\;\cdot \;log(p_{i})$$

The classification performance improvement effect of the introduced model is shown in Table 5.

After introducing the YOLO classification header, the model achieved significant improvements in classification performance. The accuracy has increased from 92.48% to 92.89%, and the F1 score has increased from 92.48% to 92.89%. The overall improvement of various indicators is about 0.41 percentage points, which is the largest improvement among all structural optimizations. The BCS ± 0.25 tolerance accuracy has also been improved to 98.10%, indicating that this structure enhances the robustness of the model in fine-level discrimination tasks. However, this improvement also brought significant computational and storage overhead: FLOPs increased by 15.13 M, parameter count increased from 4.01 M to 5.65 M, model size expanded to 21.9 MB, and inference latency significantly increased to 1.3398 ms, nearly doubling compared to the original structure.

Overall, the YOLO classification head effectively improves the expression and discriminative performance of the model through its powerful local perception and feature reuse capabilities, especially suitable for BCS-level recognition tasks with high accuracy requirements. But in resource constrained scenarios, the additional computational and storage costs need to be balanced. If the deployment environment allows, this module is an effective structural enhancement solution to improve model performance.

**Figure 9:**
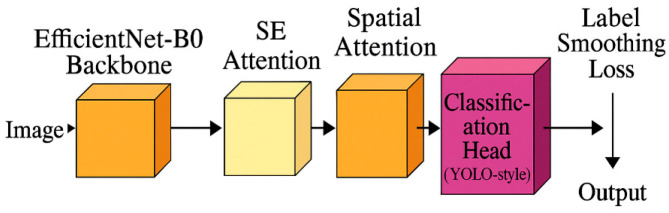
Overall Model Diagram.

Based on the above improvement methods, the final model architecture is shown in Figure 9. This model is based on the EfficientNet-B0 backbone network and integrates channel attention (SE module) and spatial attention mechanisms to enhance the model’s attention ability in the feature extraction process. In addition, introducing a label smoothing loss function effectively alleviates overfitting and improves the generalization performance of the model. Finally, using YOLO’s classification head instead of the traditional fully connected layer significantly reduces the number of model parameters while ensuring classification accuracy. The overall design achieves a good balance between precision and lightweight, suitable for practical application scenarios that require both performance and efficiency.

### 3.3. Model Recognition Performance Test

#### 3.3.1. The Confusion Matrix of the Model

**Figure 10:**
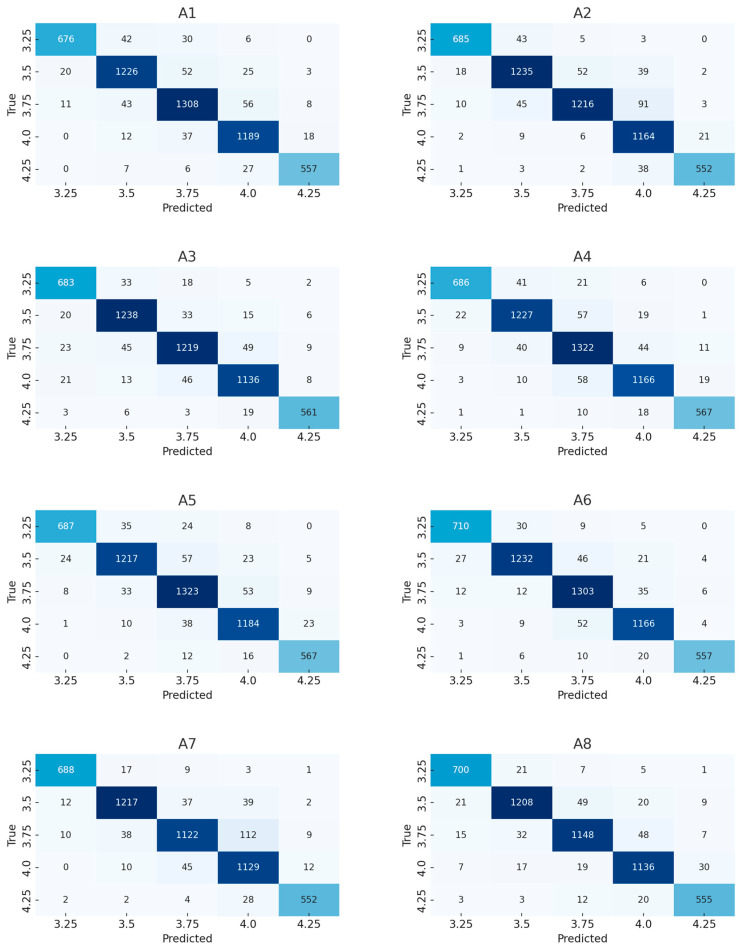
Confusion matrix diagram of ablation test. The shades of color in the figure represent the model’s prediction results for each category. Dark colors (dark blue) indicate that the model has made accurate predictions in that category, with a higher number of correctly classified samples; light colors indicate that the model’s predictions were inaccurate, with a higher number of incorrectly classified samples. Through this color difference, we can visually see in which categories the model performed well and which categories had poorer prediction results.

The confusion matrix in Figure 10 not only visually demonstrates the classification performance of the model in various categories, but also helps identify the weaknesses of the model in specific categories, such as easily confused category pairs.

**Figure 11:**
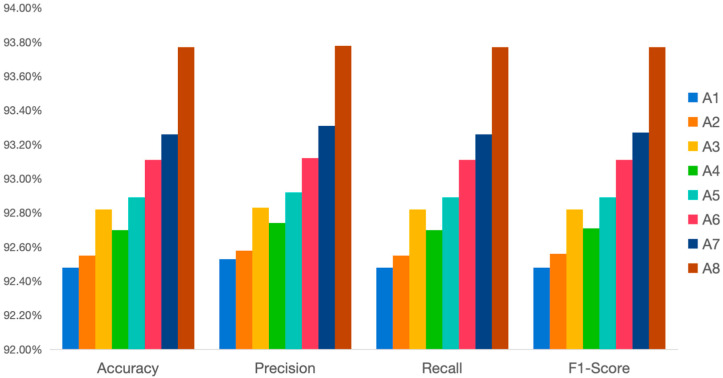
Comparison diagram of ablation test models.

Figure 11 is a chart listing some important indicators for selecting the basic model, using accuracy, precision, recall, and F1 score as the main evaluations.

#### 3.3.2. Model Ablation Test

In order to evaluate the impact of different modules and design choices on the physical condition scoring task and optimize the model structure. By gradually removing various components from the model, such as SE attention module, Spatial attention module, and label smoothing loss function, the actual contribution of each module can be clarified, ensuring that only the parts that have a significant impact on performance improvement are retained in the model. In addition, ablation experiments also help improve model accuracy while avoiding overfitting, and enhance the model’s generalization ability while maintaining computational efficiency. This experiment introduced multidimensional indicators to systematically evaluate the performance of the model, including conventional classification performance indicators (Accuracy, Precision, Recall, F1 Score), model structure indicators (Model Size, FLOPs, parameter count), inference efficiency indicators (Latency), as well as tolerance evaluation indicators of ±0.25 and ±0.5 set for BCS-level classification tasks, to comprehensively examine the accuracy, efficiency, and fault tolerance of the model in practical applications. The experimental results are shown in Table 6.

In this comparative experiment, we systematically evaluated the performance improvement effect of various structural improvement and optimization strategies on the bovine BCS model. Eight sets of experiments (A1–A8) were set up, each gradually introducing different modules on the basic EfficientNet-B0 model to achieve the dual goals of slim and performance enhancement. By comparing the changes in various experimental indicators, not only were the independent and combined contributions of each module quantified, but the rationality and effectiveness of the designed structure were also verified.

As the original model, A1 has achieved an accuracy of 92.48% and a BCS ± 0.25 accuracy of 97.98% in five classification tasks without adding any attention mechanism or optimizing the loss function, demonstrating the strong light image recognition ability of EfficientNet-B0. However, its discriminative power is still limited when dealing with feature overlap or fuzzy boundary samples (such as BCS 3.75 vs. 4.0).

After adding SE channel attention mechanism in A2, the model accuracy and F1 score were slightly improved to 92.55% and 92.56%, respectively, and the delay was reduced, indicating that the SE module has a good cost-effectiveness ratio in enhancing feature selectivity. After introducing spatial attention in A3, the improvement was even more significant, with F1 score increasing to 92.82% and ±0.25 tolerance accuracy reaching 98.04%. This indicates that the spatial-level weighting mechanism can better guide the model to focus on discriminative regions such as tail roots, strengthening the understanding and discrimination ability of spatial features.

In the A4 experiment, Label Smoothing was used to replace the original cross entropy loss, which effectively alleviated the over fitting of the model to the training data labels, and the accuracy rate of ±0.25 tolerance was increased to 98.28%, which was the best among all single modules, indicating that this loss function has obvious advantages in processing noise labels and fuzzy samples with category boundaries. A5 adopts YOLO classification head instead of traditional fully connected classification structure, which brings the largest performance improvement among all structures (Accuracy + 0.41 pp, F1 + 0.41 pp), and FLOPs also significantly increase to 415.52 M. Although the number of parameters and inference delay have increased (delay up to 1.3398 ms), its performance benefits have practical value for deployment environments with relatively sufficient computing resources.

After jointly applying SE and Spatial attention in A6, the accuracy of the model was improved to 93.11%, with accuracies of 98.28% and 99.76% under ±0.25 and ±0.5 tolerances, respectively. This indicates that the two attention mechanisms complement each other in the channel and spatial dimensions, strengthening the joint modeling ability of local features and global semantics. A7 introduces Label Smoothing on the basis of A6, which further increases F1 score to 93.27%, with little change in inference overhead, reflecting the good synergistic effect of Label Smoothing and attention mechanism.

The final A8 experiment combined all optimization modules, achieving an accuracy, precision, recall, and F1 score of 93.77%. The ±0.25 tolerance accuracy reached 98.19%, approaching the theoretical optimal value. Although the model volume has increased to 23.8 MB and the inference delay has increased to 0.9752 ms, it still remains within an acceptable range for edge deployment, balancing high accuracy and strong real-time performance.

### 3.4. Model Lightweighting Processing

Model distillation, as an efficient knowledge transfer technique, significantly reduces computational costs and enhances deployment flexibility by compressing the generalization ability of complex teacher models into lightweight student models. Hinton et al. [50] first proposed a knowledge distillation framework, which uses the output distribution softened by temperature parameters to guide student model training, laying a theoretical foundation for subsequent research. The FitNets method proposed by Romero et al. [51] has pioneering significance in the specific improvement direction of intermediate layer feature distillation. They proposed improving student model performance through intermediate layer feature matching, which is suitable for deep model compression scenarios. Sanhe et al. [52] further validated the effectiveness of this technique in pre-trained language model compression, reducing BERT’s parameter count by 40% while retaining 97% of its performance through distillation. TinyBERT, proposed by Jiao et al. [53], introduces intermediate-level feature alignment in both general pre-training and task-specific fine-tuning stages through a two-stage distillation strategy, achieving significant improvement in student model performance on natural language understanding tasks. These studies have jointly promoted the extensive application of model distillation technology in edge computing, mobile deployment, and other scenarios [54].

To achieve the deployment and application of the model in practical aquaculture scenarios, it is necessary to minimize the computational resource consumption and volume of the model while ensuring the accuracy of scoring. In response to the problem of large parameter count and slow inference speed in the original teacher model in this study, this paper chooses Knowledge Distillation as the compression method. Table 7 shows the comparison results of the model before and after distillation.

After introducing model distillation technology, the student model significantly reduced model resource overhead while maintaining high classification performance. Although the accuracy decreased from 93.77% to 91.10% and the F1 value decreased by about 2.67 percentage points, its BCS accuracy within a tolerance range of ±0.5 still improved to 99.72%, slightly higher than the teacher model’s 99.63%, indicating that its fault tolerance ability is still excellent in practical applications. In terms of resource efficiency, the FLOPs of the distilled model decreased from 481.07 M to 312.92 M, the parameter count decreased from 5.86 M to 2.23 M, the model volume was reduced to 8.7 MB, and the inference delay was also reduced to 0.8711 ms, significantly improving the edge deployment adaptability of the model. To sum up, although distillation has brought some precision loss, it has significantly reduced the burden of computing and storage while retaining the main classification capability. It is a key means to achieve light deployment, and is suitable for the intelligent scoring task of cow body condition in edge computing environment. Greatly improved deployment efficiency and resource utilization. From the experimental results, it can be seen that the distilled student model can still achieve accuracy close to the teacher model while maintaining a small model size, with higher inference efficiency and deployment adaptability, suitable for practical application in edge devices or resource constrained environments.

### 3.5. Model Interpretability Analysis (Grad CAM)

In the study of model interpretability, Grad CAM and its improved methods (such as Grad CAM++) provide intuitive decision basis explanations for deep neural networks through gradient weighted visualization techniques. The original Grad CAM proposed by Selvaraju et al. [55] reveals the model’s attention area by generating class activated heat maps, while Grad CAM++ by Chattopadhay et al. [56] further optimizes the localization accuracy in multi-objective scenarios; In safety critical areas such as autonomous driving, Kim and Canny validated the causal dependency of the Grad CAM model on key features such as lane markings and traffic signs by applying it to an end-to-end driving model, providing an important practical example for the implementation of explainable AI in the industry [57].

To further validate the rationality and interpretability of the model decisions, this paper introduces Grad CAM (Gradient weighted Class Activation Mapping) technology to visualize and analyze the attention regions of the model under different BCSs. Grad CAM can generate thermal maps of feature areas, highlighting the image regions that the model pays the most attention to during prediction, thereby revealing the basis for model judgment.

**Figure 12:**
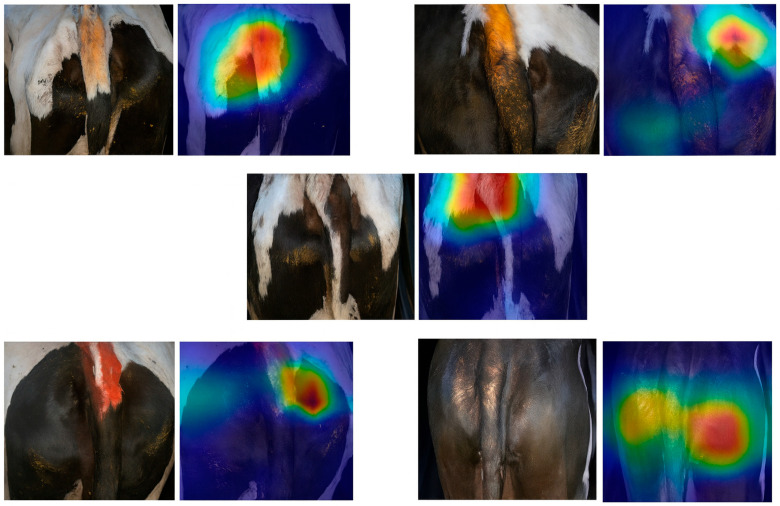
Body condition score (BCS) heatmap. The shades of color in the figure represent the degree of attention paid to different areas. The red/yellow/orange areas indicate the key areas of the model’s focus, which are usually the most important feature areas, and the activation values of the model at these positions are higher; while the blue/violet areas represent the less focused areas, and the activation values of the model at these areas are lower, indicating that they are less important in the analysis. Through this color coding, the degree of the model’s attention to different areas can be intuitively understood.

Figure 12 shows the Grad CAM visualization results of the model at five different body condition levels (BCS = 3.25~4.25). Each set of images includes the original input image and the corresponding activated heatmap. The red area represents the area that the model pays high attention to, while the blue area represents the part that the model pays less attention to. From the heat map, it can be seen that the key focus areas of the model are mostly located at the tail root position that can reflect the condition of the cow, indicating the effectiveness of the algorithm recognition.

## 4. Discussion

### 4.1. Benchmark Model Selection

This study proposes a lightweight BCS model based on cattle tail characteristics, achieving an accuracy rate of 93.77% in the BCS task. The model’s accuracy reached 98.19% under ±0.25 tolerance and 99.63% under ±0.5 tolerance, demonstrating its strong potential for deployment on edge devices. Unlike traditional deep learning-based methods, which require high computational costs, this model efficiently balances accuracy and low computational load, making it ideal for resource-constrained environments. Additionally, by applying model distillation, the model size was reduced from 23.8 MB to 8.7 MB, and the inference speed improved by 10.7%, further enhancing its adaptability on edge devices. These results align with the study’s objective of providing an efficient, real-time cattle health monitoring solution for large-scale farming.

### 4.2. SE and Spatial Attention

The EfficientNet-B0 architecture used in this study integrates SE and Spatial Attention mechanisms, significantly improving the model’s accuracy. The attention mechanism particularly boosts performance in recognizing small targets, such as the tail root area. This approach sets the model apart from previous studies like those of Lee et al. and others, which often require high hardware support due to their heavy computational demands [12,30]. The addition of attention mechanisms allows the model to more effectively capture key areas, enhancing its accuracy and efficiency, particularly in small, hard-to-detect features. Compared to other lightweight models such as ShuffleNet, this model not only performs better in accuracy but also manages resources more effectively [26].

### 4.3. Label Smoothing Loss

To mitigate overfitting and enhance the model’s generalization ability, Label Smoothing Loss was incorporated into the model’s training process. This technique reduced the model’s overconfidence in class labels, which is especially important in datasets with noisy or imbalanced categories. By smoothing the target labels, the model’s performance improved, ensuring more reliable predictions in real-world applications where tail characteristics can vary. This aligns with the study’s objective of improving model robustness and performance under real-world conditions.

### 4.4. YOLOv5 Head and Ablation

The inclusion of the YOLOv5 head for localization tasks enabled the model to focus on specific areas of interest, such as the tail, improving its ability to identify and assess cattle body condition. Ablation studies were used to evaluate the contributions of various components, such as the attention mechanism and label smoothing. The findings confirmed the importance of these components in enhancing model performance. These studies also revealed that the combination of these strategies contributed to the model’s superior accuracy compared to traditional methods, thus directly supporting the study’s goal of improving automated health monitoring systems.

### 4.5. Limitations and Future Research

Despite the promising results, this study has several limitations. First, the dataset used was sourced from a specific region in China, which may introduce geographical bias. Cattle health, environmental conditions, and breed differences could influence tail characteristics, affecting the model’s generalization across different regions. Future studies should focus on expanding the dataset to include diverse regions and cattle breeds to enhance the model’s robustness. Moreover, while the tail characteristics were useful in assessing cattle health, abnormal tail shapes due to illness or injury might reduce the model’s accuracy. To address this, incorporating additional body features, such as images of the head or back, could improve the model’s robustness. Incorporating multimodal data could further enhance the accuracy and reliability of the system [58].

Another challenge arises from the fact that the image data in this study was captured in a relatively ideal environment, whereas real-world conditions, such as variable lighting and background noise, could affect image quality and the model’s performance. Future work should focus on testing the model in more complex, real-world environments to assess its stability across different conditions. Furthermore, the model distillation process could be further optimized to reduce model size and inference time, improving deployment efficiency on edge devices. Future improvements in pruning, quantization, and other distillation techniques will enable better performance in more dynamic and resource-limited environments [59,60].

This research provides strong support for the development of intelligent agricultural technologies, especially in the areas of precision breeding and real-time health monitoring [61,62]. It demonstrates the potential of tail characteristics as a basis for BCS. With the further development of edge computing technology, the wide application of intelligent scoring systems will help improve agricultural production efficiency and promote the intelligent transformation of the agricultural industry.

## 5. Conclusions

This study proposes a lightweight BCS model for cattle, optimized for real-time deployment on edge devices. The model combines tail features and attention mechanisms to improve accuracy while maintaining low computational costs, making it suitable for resource-constrained farm environments. Model distillation further reduces size and inference latency, enhancing deployment efficiency. This solution offers an effective, scalable approach to automate cattle health monitoring, improving accuracy and efficiency in livestock management. Future research will focus on expanding the dataset, improving generalization across diverse environments, and exploring techniques like pruning and quantization to optimize model performance on edge devices, fostering the digital transformation of the livestock industry.

## Figures and Tables

**Table 1:** Comparison Table of Basic Model Performance Testing.

Metric	EfficientNet-B0	MobileNetV3-Large	MobileNetV2	MobileNetV3-Small	SqueezeNet-1	ShuffleNet
Accuracy	92.48	90.76	90.13	89.96	86.49	85.71
Precision	92.53	91.52	90.55	90.28	87.17	85.94
Recall	92.48	90.62	90.39	89.96	86.74	86.00
F1-Score	92.48	91.03	90.44	90.09	86.90	85.97
Model Size	15.31	16.63	8.7	5.9	2.8	5.0
FLOPS	400.39	224.76	312.92	58.63	263.06	147.79
Parameters	4.01	4.21	2.23	1.52	0.73	1.26
Per-sample latency	0.6829	0.9772	0.9386	0.8761	0.8928	0.8834
BCS ±0.25	97.98	97.35	97.63	97.13	96.08	95.71
BCS ±0.5	99.70	99.48	99.44	99.40	99.37	99.16

**Table 2:** Improvement effect of adjacent category discrimination (BCS3 vs. BCS4) after introducing SE Attention mechanism.

Evaluation Indicators	No SE	Have SE	Increase Amplitude
Accuracy	92.48	92.55	+0.07
Precision	92.53	92.58	+0.05
Recall	92.48	92.55	+0.07
F1-Score	92.48	92.56	+0.08
FLOPS	400.39	414.14	−13.75
Parameters	4.01	4.22	+0.21
Per-sample latency	0.6829	0.6423	−0.0406
Model Size	15.31	16.38	+1.07
BCS ± 0.25	97.98	98.02	+0.04
BCS ± 0.5	99.70	99.68	−0.02

**Table 3:** Improvement effect of introducing Spatial Attention mechanism on adjacent category discrimination (BCS3 vs. BCS4).

Evaluation Indicators	No Spatial	Have Spatial	Increase Amplitude
Accuracy	92.48	92.82	+0.34
Precision	92.53	92.83	+0.30
Recall	92.48	92.82	+0.34
F1-Score	92.48	92.82	+0.34
FLOPS	400.39	403.46	+3.07
Parameters	4.01	4.08	+0.07
Per-sample latency	0.6829	0.7063	+0.0234
Model Size	15.31	15.84	+0.53
BCS ± 0.25	97.98	98.04	+0.06
BCS ± 0.5	99.70	99.70	+0

**Table 4:** Improvement effect of adjacent category discrimination (BCS3 vs. BCS4) after introducing Label Smoothing mechanism.

Evaluation Indicators	No Label Smoothing	Have Label Smoothing	Increase Amplitude
Accuracy	92.48	92.70	+0.22
Precision	92.53	92.74	+0.21
Recall	92.48	92.70	+0.22
F1-Score	92.48	92.71	+0.23
FLOPS	400.39	413.87	+13.48
Parameters	4.01	4.01	+0
Per-sample latency	0.6829	0.7124	+0.0295
Model Size	15.31	16.4	+1.09
BCS ± 0.25	97.98	98.28	+0.30
BCS ± 0.5	99.70	99.78	+0.08

**Table 5:** Improvement effect of adjacent category discrimination (BCS3 vs. BCS4) after introducing YOLO classification head mechanism.

Evaluation Indicators	No YOLO Classification Head	Have YOLO Classification Head	Increase Amplitude
Accuracy	92.48	92.89	+0.41
Precision	92.53	92.92	+0.39
Recall	92.48	92.89	+0.41
F1-Score	92.48	92.89	+0.41
FLOPS	400.39	415.52	+15.13
Parameters	4.01	5.65	+1.64
Per-sample latency	0.6829	1.3398	+0.6569
Model Size	15.31	21.9	+6.59
BCS ± 0.25	97.98	98.10	+0.12
BCS ± 0.5	99.70	99.70	+0

**Table 6:** Comparison Table of Ablation Experimental Models.

Metric	A1	A2	A3	A4	A5	A6	A7	A8
Attention module	none	SE	Spatial	none	none	SE + Spatial	SE + Spatial	SE + Spatial
Loss function	CrossEntropy	CrossEntropy	CrossEntropy	CrossEntropy	CrossEntropy	CrossEntropy	LabelSmoothing	LabelSmoothing
Describe	base model	Add SE attention module	Add Spatial Attention Module	Add label smoothing loss	Add YOLO classification header	Add combined attention	Add label smoothing loss function	Add YOLO classification header
Accuracy	92.48	92.55	92.82	92.70	92.89	93.11	93.26	93.77
Precision	92.53	92.58	92.83	92.74	92.92	93.12	93.31	93.78
Recall	92.48	92.55	92.82	92.70	92.89	93.11	93.26	93.77
F1-Score	92.48	92.56	92.82	92.71	92.89	93.11	93.27	93.77
FLOPS	400.39	414.14	403.46	413.87	415.52	403.72	403.73	481.07
Parameters	4.01	4.22	4.08	4.01	5.65	4.28	4.28	5.86
Per-sample latency	0.6829	0.6423	0.7063	0.7124	1.3398	0.6723	0.6872	0.9752
Model Size	15.31	16.38	15.84	16.4	21.9	16.6	17.4	23.8
BCS ± 0.25	97.98	98.02	98.04	98.28	98.10	98.28	98.06	98.19
BCS ± 0.5	99.70	99.68	99.70	99.78	99.70	99.76	99.72	99.63

**Table 7:** Comparison Table of Models Before and After Distillation.

Evaluation Indicators	Before Distillation	After Distillation
Accuracy	93.77	91.10
Precision	93.78	91.14
Recall	93.77	91.10
F1-Score	93.77	91.10
FLOPS	481.07	312.92
Parameters	5.86	2.23
Per-sample latency	0.9752	0.8711
Model Size	23.8	8.7
BCS ± 0.25	98.19	97.57
BCS ± 0.5	99.63	99.72

## Data Availability

The datasets used and analyzed during the current study are pub-licly available. The dataset can be accessed at https://cstr.cn/31253.11.sciencedb.16704 (accessed on 7 January 2025).
